# Determination of rheology and surface tension of airway surface liquid: a review of clinical relevance and measurement techniques

**DOI:** 10.1186/s12931-019-1229-1

**Published:** 2019-12-04

**Authors:** Zhenglong Chen, Ming Zhong, Yuzhou Luo, Linhong Deng, Zhaoyan Hu, Yuanlin Song

**Affiliations:** 1 School of Medical Instrumentation, Shanghai University of Medicine & Health Sciences, 257 Tianxiong Road, Shanghai, 201318 China; 2 Department of Intensive Care Medicine, Zhongshan Hospital, Fudan University, 180 Fenglin Road, Shanghai, 200032 China; 3 Institute of Biomedical Engineering and Health Sciences, Changzhou University, Changzhou, 213164 Jiangsu China; 4 School of Medical Instrumentation, Shanghai University of Medicine & Health Sciences, 257 Tianxiong Road, Shanghai, 201318 China; 5 Department of Pulmonary Medicine, Zhongshan Hospital Fudan University, 180 Fenglin Road, Xuhui District, Shanghai, 200032 China

**Keywords:** Airway surface liquid, Lung surfactant, Viscosity, Surface tension, Rheology, Ventilator-induced lung injury, Respiratory diseases

## Abstract

By airway surface liquid, we mean a thin fluid continuum consisting of the airway lining layer and the alveolar lining layer, which not only serves as a protective barrier against foreign particles but also contributes to maintaining normal respiratory mechanics. In recent years, measurements of the rheological properties of airway surface liquid have attracted considerable clinical attention due to new advances in microrheology instruments and methods. This article reviews the clinical relevance of measurements of airway surface liquid viscoelasticity and surface tension from four main aspects: maintaining the stability of the airways and alveoli, preventing ventilator-induced lung injury, optimizing surfactant replacement therapy for respiratory syndrome distress, and characterizing the barrier properties of airway mucus to improve drug and gene delivery. Primary measuring techniques and methods suitable for determining the viscoelasticity and surface tension of airway surface liquid are then introduced with respect to principles, advantages and limitations. Cone and plate viscometers and particle tracking microrheometers are the most commonly used instruments for measuring the bulk viscosity and microviscosity of airway surface liquid, respectively, and pendant drop methods are particularly suitable for the measurement of airway surface liquid surface tension in vitro. Currently, in vivo and in situ measurements of the viscoelasticity and surface tension of the airway surface liquid in humans still presents many challenges.

## Background

Human airways, from the trachea through the bronchioles to the alveoli, are lined on the inside with a continuous film of surface liquid, which increases in thickness from ~ 0.1 μm in the alveoli to ~ 10 μm in the trachea [1–5]. In the airways, this film of liquid is a bilayer consisting of a periciliary layer and a mucous or ‘gel’ layer atop. The mucous layer is composed of 97% water and 3% solids (mucins, nonmucin proteins, salts, and cellular debris), which determine the linear and nonlinear viscoelasticity and diffusive properties of the mucus [6, 7]. The periciliary layer is of low viscosity containing water and solutes and in which the cilia reside [8]. The cilia beat 12 to 15 times per second, with cilia tips intermittently gripping the underside of the mucous layer, thus propelling it and entrapped particles towards the mouth, where it is swallowed or expectorated. In this way, the airways are kept clean. In addition to acting as a solid physical barrier to most pathogens, the airway lining fluid also contains lysozymes and a range of defensins that are capable of chemically inactivating inhaled pathogens [1].

In airway generations beyond 15 or 16, the primary secretory cells are Club cells (originally known as Clara cells), found in the respiratory bronchioles, and type II epithelial cells, found in the alveoli. These two types of cells have a relatively weaker mucus-secreting capacity compared with the Goblet cells found in the bronchi and conducting bronchioles [1, 4]. As a result, the airway lining fluid transitions from a two-layered fluid to a single layer of fluid that is primarily saltwater, yet with significant concentrations of surfactant [9]. Pulmonary surfactant (PS) is a complex mixture of some 90% lipids and 10% proteins. Most of the lipids are phospholipids, of which 70–80% are dipalmitoyl phosphatidyl choline (DPPC), the main surface-active material responsible for lowering surface tension, while the surfactant proteins (SP) are SP-A, SP-B, SP-C and SP-D [1, 10]. Among these surfactant-associated proteins, SP-B and SP-C are vital to the stabilization of the surfactant monolayer; SP-A and SP-D are involved in the control of surfactant release and possibly in the immunology of the lung. The main function of the pulmonary surfactant film is to reduce surface tension at the air-liquid interface, thus preventing collapse of the alveoli and small airways during end-exhalation [11].

Pulmonary surfactant is present not only in the alveoli but also in the bronchioles and small airways. We hereafter refer to the thin fluid continuum consisting of the airway lining layer and the alveolar lining layer as airway surface liquid [9]. Changes in macro- and microrheological properties of airway surface liquid have a significant impact on normal respiratory mechanics and normal barrier and clearance functions of the lung [12, 13]. For example, an intermediate viscoelasticity of the mucous gel layer, or in other words a viscosity in the range of 12–15 Pa ∙ s(1 Pa ∙ s = 1000 cP) and an elastic modulus of 1 Pa, are essential for optimal mucociliary clearance [14–16]. If the viscoelasticity of airway mucus becomes too low, however, the elasticity is not enough for mucus to counteract gravitational action, which likely makes mucus to slide down into the lung and flood the alveoli [13, 17–19]. In contrast, pulmonary disease conditions, such as cystic fibrosis (CF), chronic obstructive pulmonary disease (COPD), and asthma, are usually characterized by an increase in the viscoelasticity of mucus. As a result, ciliary action and cough are incapable of effectively clearing the sticky mucus, leading to the accumulation of mucus and even the complete blockage of the airway observed in the above disorders [8, 13, 20].

Likewise, alterations in the surface tension of the alveolar lining fluid also cause severe respiratory diseases, such as neonatal respiratory distress syndrome (NRDS) and acute lung injury (ALI) or the acute respiratory distress syndrome (ARDS) [21–23]. Premature infants who suffer from NRDS due to surfactant deficiency exhibit a high lining-fluid surface tension and hence a propensity for prominent atelectasis, decreased lung compliance, increased work of breathing and impaired gas exchange. The postnatal delivery of exogenous surfactant can significantly lower surface tension forces in the lung and has been established as a standard therapeutic intervention in the management of preterm infants with NRDS [24]. Similarly, in ALI/ARDS patients, the normal function of PS is inhibited by protein-rich oedematous fluid present in the airspaces, leading to a dramatic increase in surface tension and hence a decrease in lung compliance [25]. The development of ventilator-induced lung injury (VILI) in mechanically ventilated ALI/ARDS patients is largely attributed to this change in the surface tension-relevant lung micromechanics.

Therefore, the study of the rheological properties of airway surface liquid has both physiological and clinical significance. Unfortunately, due to a lack of suitable in vivo and in situ measurement techniques, thus far, all rheological measurements of human respiratory mucus came from in vitro studies that may not give a true picture of in vivo conditions. Moreover, reported literature values of the viscoelasticity of human respiratory mucus show large (orders of magnitude) intersubject, intrasubject, and even within the same mucus -sample variations (Table 1). It is imperative to heighten collaborations between clinicians, biomedical engineers, and applied scientists to explain these variations in perspective of both physiology and experimental techniques, to further develop tools to assess the quantitative properties of airway surface liquid, and finally to correlate the biophysical properties of airway surface liquid with healthy versus diseased states. This article reviews the importance of airway surface liquid rheology and surface tension measurements in: (1) maintaining the stability of small airways and alveoli; (2) preventing ventilator-induced lung injury; (3) optimizing surfactant replacement therapy (SRT); and (4) characterizing lung barrier and clearance functions. Subsequently, new methods and techniques for determining the viscosity and surface tension of airway surface liquid are described.

**Table 1:** Comparison of Published Viscosities of Airway Surface Liquid

Reference	Viscosity (cP)	Device	Technique
Human (recurrent bronchitis) (Puchelle et al. 1981) [26]	2.48 × 10^4^	Concentric cylinder rheometer	Shear deformation at a shear rate of 0.3 *s*^−1^
Human (mild chronic bronchitis) (Puchelle et al. 1981) [26]	1.14 × 10^4^	Concentric cylinder rheometer	
Human (severe chronic bronchitis) (Puchelle et al. 1981) [26]	1.25 × 10^4^	Concentric cylinder rheometer	
Human (Baconnais et al. 1999) [27]	200	Cone and plate rheometer	Creep-test under a constant stress of 10 Pa
Human (CF) (Baconnais et al. 1999) [27]	600	Cone and plate rheometer	
Human (Jeanneret-Grosjean et al.1988) [16]	(1.2~1.5) × 10^4^	Magnetic microrheometer	Oscillating a steel microsphere at 1 and 100 rad/s
Human (CF) (Dawson et al. 2003) [28]	~ 7 × 10^4^	Cone and plate rheometer	Shear deformation at a shear rate of 10^− 2^~10^2^ rad/s
Human (CF) (Feather et al. 1970) [29]	21~134	Cone and plate rheometer	Shear deformation at a shear rate of 900 *s*^−1^
Human (chronic bronchitis) (Feather et al. 1970) [29]	117~144	Cone and plate rheometer	
Human (bronchiectasis) (Feather et al. 1970) [29]	58	Cone and plate rheometer	
Human (ARDS) (author’s labs)	0.97~ 7.76 × 10^4^	Cone and plate rheometer	Shear deformation at a shear rate of 10^− 2^~10 rad/s

*cP:* centipoise, *1 cP =* 0.001 Pa·s, *CF:* Cystic fibrosis

## Importance of rheological measurements of airway surface liquid

### Maintaining stability of airways and alveoli

**Fig. 1:**
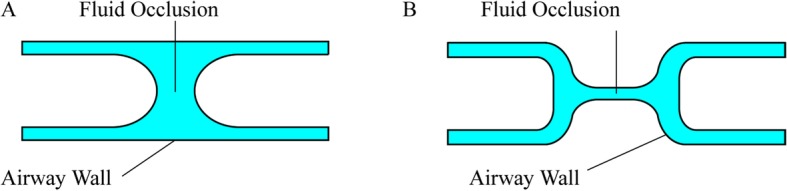
Mechanisms of airway closure: (A) Liquid bridge formation (B) compliant collapse

The role of the surface tension and viscosity of airway surface liquid in maintaining airway stability is primarily two-fold: retarding small airway closure and preventing alveolar collapse. As described in Section 1, the liquid lining usually forms a thin and relatively uniform layer on the inner surface of the airway, but sometimes it is possible for the airway to become occluded by the liquid, leading to airway closure. Closure of the distal airways at low lung volumes can occur through two mechanisms, “liquid bridge formation” or “compliant collapse” (Fig. 1) [30–32]. In the former case, liquid in a uniform film lining on the inner wall of an axisymmetric airway redistributes via a classical fluid-elastic instability known as the Plateau-Rayleigh instability [30, 33, 34]. This process leads to complete occlusion of the airway by a liquid plug or “bridge”, provided that the condition of sufficient liquid for closure is satisfied. A theoretical analysis by White and Heil showed that the growth rate of the film thickness increased with surface tension and decreased with an increase in the fluid’s viscosity [35]. Halpern and coworkers revealed that the growth rate for a viscoelastic layer was larger than for a Newtonian fluid with the same viscosity [36]. The overall timescale required for an occlusion to form is small compared with a single breathing cycle, provided that no surfactant is present. Halpern and Grotberg further demonstrated that the closure time for a pulmonary surfactant-rich film can be approximately five times greater than that for a film free of pulmonary surfactant [37].

Alternatively, if the surface tension of the airway lining fluid is sufficiently large relative to the airway’s bending stiffness, a fluid-elastic “compliant collapse” is more likely to occur [30, 31, 33]. As lung volume falls during expiration, the radius of the airway is decreased, thus resulting in an increase of the curvature of the air-liquid interface. The initially uniform and axisymmetric liquid lining can become unstable, and pressure gradients are induced in the fluid that drive flows redistributing the fluid. As a result, in the region where the liquid lining film is thickest, surface tension creates a large pressure jump over the highly curved air-liquid interface, causing negative pressure in the liquid. At the same time, parenchymal tethering forces on the external surface of the airway fall because of the gradual increase in lung volumes. This combination of reduced lining fluid pressure and parenchymal tethering subjects the airway wall to a significant compressive load and promotes the propensity of the airway to buckle inward, producing a compliant collapse. In diseased conditions such as pulmonary oedema or neonatal RDS, this compliant collapse of the airways may occur due to an increase in the volume of fluid or in the surface tension.

**Fig. 2:**
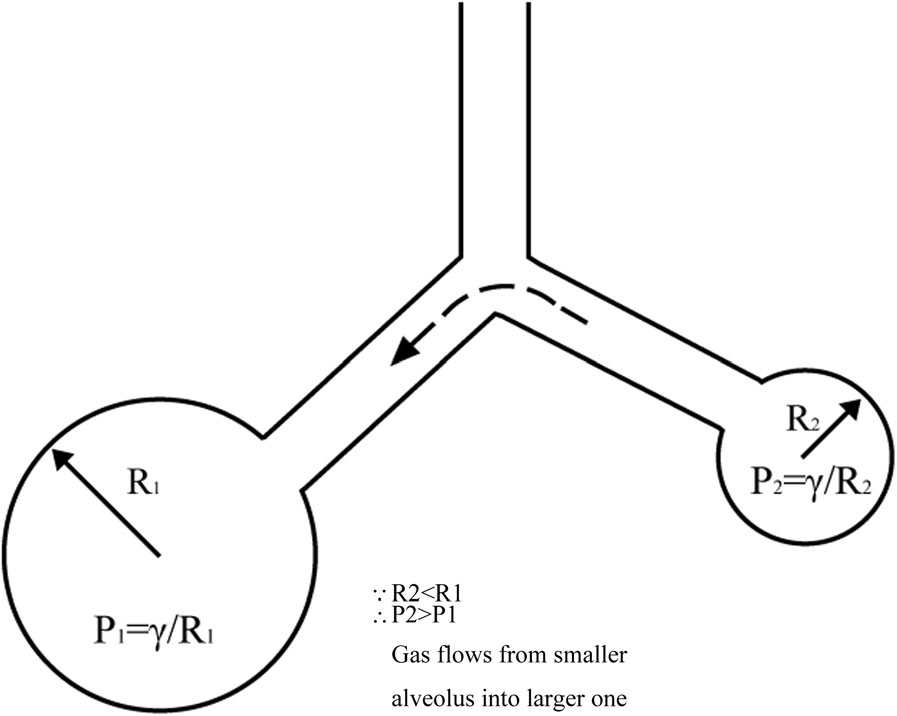
Connected alveoli illustrating the driving force collapsing the smaller alveolus in the case of constant surface tension

Surface forces also have a critical effect on airspace stability, as illustrated in Fig. 2. Two connected bubbles (alveoli) with a common pressure and a constant surface tension are blown at the end of a Y-tube [1, 38]. According to the Laplace equation, the pressure generated by surface tension in the small bubble is larger than that in one with a greater diameter, resulting in an inherently unstable system: the smaller alveolus will eventually collapse and the larger one will become over-distended. Of course, this is not the case in a healthy lung. The surface tension of the alveolar lining fluid is variable in situ as a function of expansion and compression of the alveolar surface area due to the presence of pulmonary surfactant. The surface tension drops as the alveolar surface decreases, and it rises when the surface expands, allowing for equal pressure between two different sized alveoli; therefore, system stability is maintained.

### Preventing ventilator-induced lung injury

A number of theoretical and experimental studies have demonstrated that the increase in viscosity and surface tension of airway surface liquid likely results in VILI. Two main physical mechanisms for VILI are lung tissue overdistention caused by surface tension-induced alterations in interalveolar micromechanics and atelectrauma to the epithelial cells during repetitive airway reopening and closure [39–41]. The prediction from an adjoining two-alveoli model by Chen et al. [42] shows that the pattern of alveolar expansion can appear heterogeneous or homogeneous, strongly depending on differences in air-liquid interface tension on alveolar segments. More specifically, if surface tension in the liquid-filled alveolus is much greater than that in the air-filled alveolus, then alveolar expansion is heterogeneous. Consider a pair of juxtaposed alveoli: the maximum stress and strain within the septum shared by the two alveoli may occur at a low alveolar pressure; in contrast, as alveoli inflate to near total lung capacity (TLC), the stress and strain of the alveolar walls may decrease instead. On the other hand, if the surface tensions in two adjacent alveoli are identical, then alveolar expansion is homogenous; that is, the stress and strain of all alveolar septa will appear to linearly increase as alveolar volume varies from functional residual capacity (FRC) to near TLC. These calculations are in good agreement with the experimental phenomenon observed by Perlman and coworkers [43]. Using real-time optical section microscopy, these investigators quantified the micromechanics of an air-filled alveolus that shares a septum with a liquid-filled alveolus. Instilling liquid into the alveolus produced a meniscus that changed the septal curvature and consequently the pressure difference across the septum. As a consequence, the air-filled alveolus bulged into its liquid-filled neighbour even at FRC. Given that liquid-filled and air-filled alveoli can be focal, diffuse or patchy in pulmonary oedema, these findings may provide a novel understanding of segmental heterogeneities and alveolar overdistension during mechanical ventilation.

Using thin-walled polyethylene tubes to mimic bronchial walls held in apposition by airway lining fluid, Gaver III et al. [39] investigated the effect of the tube radius (R) and the surface tension and viscosity of airway lining fluid on the airway opening velocity (U) and the applied opening pressure. They found that increasing the surface tension (γ) or viscosity (μ) resulted in an increase in airway opening pressures. Gaver III et al. further defined a nondimensional parameter capillary number (Ca ≡ *μU*/*γ*) to represent the relative importance of viscous and surface tension forces in airway opening. When Ca is small, the applied opening pressure must exceed the “yield pressure” ~8γ/R before airway opening can proceed. When Ca is larger than 0.5, the contribution of viscous forces to the overall opening pressures is non-negligible.

In subsequent studies, Bilek and Kay et al. utilized a parallel-plate flow chamber lined with pulmonary epithelial cells as an idealized model airway to investigate the mechanisms of surface tension-induced epithelial cell damage [44]. The narrow channel of the chamber was filled with either phosphate-buffered saline (high surface tension) or Infasurf (ONY, Buffalo, NY), a biologically derived pulmonary surfactant with low surface tension. Airway reopening was generated by the steady progression of a semi-infinite bubble of air along the length of the channel, which displaced the occlusion fluid. Two bubble progression velocities were investigated, and the results showed that for the saline-occluded channels, both slow and fast bubble velocities resulted in significant cellular injury compared with the control and that for the Infasurf-occluded channels, cellular injury was dramatically reduced at both bubble velocities, indicating that surfactant has a protective effect. A comparison of the experimental and theoretical observations demonstrated that among four potentially injurious components of the stress cycle associated with airway reopening (shear stress, pressure, shear stress gradient or pressure gradient), the pressure gradient was the most predominant mechanism underlying the observed cellular damage [45].

Recently, Chen et al. [46] further estimated in situ the magnitudes of mechanical stresses exerted on the alveolar walls during repetitive alveolar reopening by using the tape-peeling model from McEwan and Taylor [47]. Their calculations showed that 1) for a lung with normal fluid viscosity and surface tension, the predicted maximum shear stresses were less than 15 dyn/cm^2^ at all alveolar opening velocities that were in the physiological range, whereas for a lung with an elevated viscosity of the alveolar lining fluid, shear stresses may increase by several orders of magnitude, enough to induce epithelial cell injury; 2) similarly, in the case of elevated viscosity, pressure drops across a cell may rise to levels greater than 300 dyn/cm^2^ and consequently result in hydraulic epithelial cracks [48]; 3) the capillary pressure for alveolar opening ranged from 5 to 30 cmH_2_O, strongly depending on the initial depth of the alveolar lining fluid, which may explain the clinically high opening pressure in sticky atelectasis; and 4) assuming alveolar lining fluid to be a Newtonian flow, the magnitudes of shear stress were proportional to the alveolar opening velocity, and therefore, the reduction of inspiratory flow rate or respiratory frequency would lead to a decrease in shear stress and a concomitant reduction in atelectrauma on alveolar epithelial cells.

As discussed above, rheological measurements of airway surface liquid during the progression of the disease in many pulmonary disorders have an important role in the management of mechanical ventilation. Measured values of surface tension and viscoelasticity provide clinical data for establishing and validating mathematical models of VILI. Knowing the values of viscosity and surface tension of airway surface liquid enables clinicians to quickly determine individualized inflation pressures and PEEPs and to roughly estimate lung stress and strain based on computational models, thereby adjusting the ventilation settings or therapeutic strategies in time to avoid VILI as much as possible.

### Optimizing surfactant replacement therapy

Exogenous SRT has been established as a standard therapeutic intervention for preterm and term neonates with clinically confirmed respiratory distress syndrome since the early 1990s [10, 21, 49]. During traditional SRT, natural or synthetic surfactant is administered via an endotracheal tube either as a bolus or by infusion via a thin catheter inserted into the endotracheal tube. Thereafter, the infants are maintained on mechanical ventilation. The INSURE (INtubtion-SURfactant-Extubation) technique, which features early bolus instillation of surfactant with prompt extubation to nasal CPAP, has also been studied in a number of small randomized trials. The results showed that this strategy reduced the need for mechanical ventilation and improved survival rates [21, 50, 51]. A number of alternatives to the administration of surfactant include the use of aerosolized surfactant preparations, laryngeal mask airway-aided delivery of surfactant, instillation of pharyngeal surfactant, and administration of surfactant using laryngoscopy or bronchoscopy [21, 51].

SRT has also been applied to adults whose surfactant systems are compromised by ARDS, but clear indications of a distinct surfactant-mediated decrease in mortality or improvement in ventilator care of ARDS patients are still lacking [49, 52]. Additionally, recent randomized clinical trials have indicated that preventive surfactant administration to infants with suspected NRDS is no longer effective in groups of infants when CPAP is used routinely [21]. Most previous studies on SRT failures have focused on examining the biophysical mechanisms for surfactant inhibition due to plasma proteins or lipids [49]. However, the three-dimensional model of SRT recently proposed by Filoche and colleagues provides new insights into this issue, as it strongly suggests that inadequate delivery of surfactant may be a major cause of SRT failure [53]. Using similar surfactant mixtures and instilled dose volume, these investigators simulated the delivery of surfactant to neonates and adults in 3D structural models of the lung airway tree. The results revealed well-mixed distributions in the neonatal lungs but very inhomogeneous distributions in the adult lungs.

When liquid surfactant mixtures are instilled into the trachea via an endotracheal tube, they form liquid plugs, which are then blown distally into the branching network of the airways by forced inspirations. Filoche and colleagues simplified the above complicated flow process into two separate steps: step A, deposition of the liquid onto the airway walls into a trailing film; and step B, liquid plug splitting at an airway bifurcation. Step A determines the total amount of liquid reaching the acini, i.e., the delivery efficiency. Theoretical work by Helpern et al. [54] has shown that the thickness (h) of a trailing film in the parent tube is related to the local capillary number by the relation

1 $$ \frac{\mathrm{h}}{{\mathrm{a}}_1}=0.36\left(1-{\mathrm{e}}^{-2{\mathrm{Ca}}_{\mathrm{p}}^{0.523}}\right) $$

where a_1_ is the radius of the parent tube, the capillary number Ca_p_ = μU_p_/γ represents the ratio of viscous force (μ) to surface tension force (γ), and U_p_ is the plug speed. Eq. (1) shows that as the viscosity of the liquid plugs or the airflow rate increases, so does *Ca*_*p*_, and thus there is more liquid deposited into the trailing film. Step B governs the homogeneity of delivery. When the liquid plug splits at the bifurcation of the airway, a fraction of the plug’s volume goes down one daughter airway, *V*_1_, and the rest goes down the other, *V*_2_. Zheng and coworkers [55, 56] defined the ratio of the volumes in the daughter airways as the split ratio, *R*_*s*_ = *V*_1_/*V*_2_, which is affected by a number of factors, including the physical properties of the liquid (viscosity, density and surface tension), the gravitational orientation, the airway geometry, plug propagation speed, interfacial activity, and the presence of plug blockage in nearby airways from previous instillations. A critical parent tube capillary number was found to exist below which *R*_*s*_ = 0, and above which *R*_*s*_ increased and eventually levelled out with *Ca*_*p*_. This feature can be explained by the driving pressure at the bifurcation. When the fluid viscosity or plug velocity is too small, the driving pressure is not large enough to overcome gravity; thus, no liquid enters the upper (gravitationally opposed) daughter airway after bifurcation.

In summary, the viscosity and surface tension of surfactant mixtures have a profound effect on the distribution quality of the delivered surfactant. For example, a computational model of SRT by Filoche and colleagues showed that the synthetic surfactant mixture Exosurf, with a low viscosity of ~ 3 cP, yielded a less homogenous distribution compared with the surfactants Survanta, Curosurf and Infasurf, each with a viscosity of ~ 30 cP, under the same neonatal treatment protocol. Furthermore, in the distal regions of the lung, surface tension gradient-induced Marangoni flows drive the surfactant deeper into the lung. This requires that the surface tension of the endogenous surfactant be above that of the instilled exogenous surfactant [57].

### Characterizing barrier properties of mucus

The airway mucus gel layer acts as a solid physical barrier to foreign pathogens, toxins and environmental ultrafine particles while allowing rapid passage of selective small molecules, ions, capsid viruses and many proteins. These selective barrier properties of airway mucus are intimately related to its viscoelasticity, which shows order-of-magnitude variations in healthy versus diseased states. The rheological characterization of airway mucus has contributed greatly to both the understanding of mucocilliary clearance and the quantitation of the severity of airway diseases such as CF, COPD and chronic bronchitis [13].

For example, Hill et al. reported that the mean solids concentration (% solids by weight including salts, denoted wt%) of sputum for normal subjects is 1.7%, whereas the sputum wt% of COPD and CF subjects are ~ 2× higher (3.5%) and ~ 4× higher (7.0%), respectively. Below 3.0 wt%, the loss (viscous) modulus of human bronchial epithelial (HBE) cell culture mucus dominates the storage (elastic) modulus across all frequencies ranging from ~ 0.1 Hz (tidal breathing frequency) to ~ 10 Hz (ciliary beating frequency), implying that low wt% mucus is a viscoelastic solution; at 4.0 wt%, the elastic and viscous moduli were almost equal over the above frequency range, suggestive of the beginning of a transition from solution-like to gel-like behaviour; and at 5.0 wt%, the elastic modulus dominated the viscous modulus, suggesting that high solids wt% mucus is a viscoelastic gel at frequencies above 0.1 Hz [58]. These findings provide key data linking increased mucus solids concentration to the observation of Puchelle and Zahm [59] that when cilia beat against a fluid viscosity of higher than 100 cP, the ciliary beating frequency decreases and mucus clearance slows. In addition, Button and coworkers recently found that mucus concentration was also strongly correlated with the mucus-epithelial surface adhesive and mucus cohesive strengths. The increased mucus concentration and viscous energy dissipation in CF and COPD patients therefore make the cough mechanism fail to effectively clear accumulated mucus from the lungs [60].

The gel-on-brush model of the mucus clearance system by Button et al. demonstrated that the mucus and pericilliary brush layers must be in relative osmotic modulus balance for effective mucociliary clearance [20]. At a mucus solids concentration of ~ 5 wt% or above, the corresponding osmotic modulus of the mucus layer begins to exceed that of the PCL, therefore collapsing the PCL and slowing down the mucus clearance observed in diseases. Kesimer et al. tested the relationships predicted by the gel-on-brush model between total mucin concentration and the increase in severity of chronic bronchitis [61]. The mean total mucin concentrations were higher in current or former smokers with severe COPD than in controls who had never smoked. The relationships between total mucin concentration and prospective annualized respiratory exacerbation showed that mucin concentrations were higher in participants who had exacerbations than in those who had none. These results suggest that airway mucin concentrations may serve as a biomarker for the diagnosis of chronic bronchitis.

Microrheology affords a detailed characterization of the barrier properties of airway mucus at a scale relevant to pathogens, toxins, and foreign particles. When the scale approaches the mesh size of the mucus layer, the diffusion rates of particles are expected to be reduced due to steric or adhesive forces, thus leading to a higher apparent viscosity. A variety of conventional nanoparticle-based drug delivery systems for CF and other pulmonary diseases have been discouraged by the mucus barrier since nanoparticles are usually subjected to mucociliary clearance before they reach airway mucosal surfaces due to the extremely slow diffusion rates of these particles in the mucus. As such, to engineer nanoparticles capable of penetrating this highly viscoelastic and adhesive mucus barrier, it is imperative to characterize the local viscoelasticity of mucus at scales relevant to nanoparticle delivery systems. Suk and coworkers investigated the effect of nanoparticle size and surface chemistry on transport rates in fresh, undiluted CF sputum. They found that the transport rates of 200 nm particles that were densely coated with low molecular weight polyethylene glycol (PEG) were 90-fold faster than the same-sized uncoated particles. On the other hand, the movement of both coated and uncoated 500 nm particles was strongly hindered. Therefore, by tracking the displacement of 200 nm PEG-coated particles, they further showed that the local microviscosities of fresh, undiluted cystic fibrosis sputum were only 5-fold higher than the viscosity of water, while the bulk viscosity was 10,000-fold higher at low shear rates. Additionally, these authors estimated the average mesh spacing of physiological human CF sputum to be 140 ± 50 nm [7]. In light of these findings, investigators have further designed various muco-inert nanoparticles that can rapidly penetrate the mucus layer, thus enhancing the efficacy of drug and gene delivery at mucosal surfaces [62, 63].

## Techniques for measuring the rheology of airway surface liquid

When selecting an appropriate technique to investigate the viscosity of airway surface liquid, it is important to keep in mind that airway surface liquid has two particular features: 1) a relatively small available sample volume and 2) large variations in the range of viscosity depending on the patient, the sampling site in the lung, and healthy or diseased conditions [7, 13–16, 26, 27, 29, 59, 64]. Commonly used instruments for the measurement of viscosity include glass capillary viscometers, falling sphere viscometers, rotational viscometers, magnetic microrheometers and particle tracking microrheometers. Among these instruments, the first four have been used to determine the macroscopic bulk viscosity, while the last one has been applied to the study of microrheology.

### Glass capillary viscometers

Glass capillary viscometers are also known as tube-type viscometers, which consist of a U-shaped glass tube with a reservoir bulb on one arm of the U and a measuring bulb with a precise narrow bore (the capillary) on the other. There are two calibrated marks along the length of the capillary. During use, liquid is suctioned into the measuring bulb and then allowed to flow downward through the capillary into the reservoir. The time it takes for the liquid to pass between two marks is a measure of the viscosity η [65]. The principle of the glass capillary viscometer is based on the Poiseuille law [66]:

2 $$ \eta =\frac{\pi {r}^4\varDelta Pt}{8 LV} $$

where *r* is the radius of the capillary tube, *ΔP* is the pressure difference between the two ends of the capillary tube, t is the time it takes for a volume V of fluid to elute, and L represents the length of the capillary tube. Theoretically, the more viscous the liquid, the longer it takes to flow.

Basch et al. first used a capillary viscometer to measure sputum viscosity, but their results were later demonstrated to be unreliable by Forbes and Wise [67, 68]. Despite a variety of modified versions later, using a wide-bore or horizontal tube, for instance, the measurements with sputum were still widely scattered and not reproducible, as the sputum frequently either slipped through the tube as a solid plug or remained stuck somewhere in the tube. In addition, lung fluids such as mucus and sputum behave as a non-Newtonian viscosity that is dependent on shear rate. Thus, capillary viscometers are further limited since they can only measure viscosity for one shear rate at a time.

### Falling-sphere viscometers

Stokes’ law is the basis of the falling-sphere viscometer, in which the fluid under examination is stationary in a vertical or inclined glass tube. A small sphere is allowed to move through the test fluid. As the falling velocity of the sphere increases, the frictional force also increases, and eventually, a terminal velocity Vs is reached when the gravitational force is balanced with the buoyant force and this frictional force. The viscosity η of the test fluid can be calculated by Stokes’ law:

3 $$ \eta ={d}^2\left({\rho}_s-{\rho}_f\right)g/18{V}_s $$

where d is the sphere diameter, *ρ*_*s*_ is the sphere density, *ρ*_*f*_ is the fluid density, and g is the local gravitational acceleration [69].

Falling sphere viscometers have undergone important modifications over the years; some commercially available instruments, for example, use cylindrical needles or pistons with hemispheric ends instead of spheres [65]. Unlike the traditional falling sphere viscometer that only applies for viscosity measurements of Newtonian fluids, the falling needle also possesses the ability to measure non-Newtonian rheological parameters [70]. In terms of sputum viscosity, there are several drawbacks associated with the falling sphere viscometers, including the requirement of a significant sample volume, operation at low shear rates and poor measurement stability and reproducibility. For instance, Elmes and White measured sputum viscosity employing a rolling ball viscometer and found that the ball moved along the line of least resistance and rolled around the aggregation of viscous material suspended in the sputum [71].

### Rotational viscometers

Rotational viscometers use the concept that viscosity is defined as the ratio of shear stress to shear rate. They measure the torque required to rotate an immersed element (the spindle) in a fluid at a known speed. The spindle is driven by a motor through a calibrated spring. By utilizing a multiple speed transmission and interchangeable spindles, a wide range of viscosities can be measured, thus enhancing the versatility of the instruments.

There are two basic types of rotational viscometers, one with two coaxial cylinders and the other with a cone and plate [65, 66]. In the cylinder viscometer, the liquid to be tested is placed in a narrow space between the rotating cylinder and the fixed cylinder. The more viscous the fluid is, the greater the torque required to spin the rotating cylinder. The primary disadvantage of the cylinder viscometer is the relatively large sample volumes required. For example, despite the use of specifically designed Small Sample Adapter, commercial Wells-Brookfield cylinder viscometers still require a sample volume of 2 to 16 mL. Baldry and Josse found that the rotating cylinder did not move at all or rotated with a very low speed when sputum viscosity was relatively high [66]. For these reasons, they are not extensively used in clinical laboratories.

**Fig. 3:**
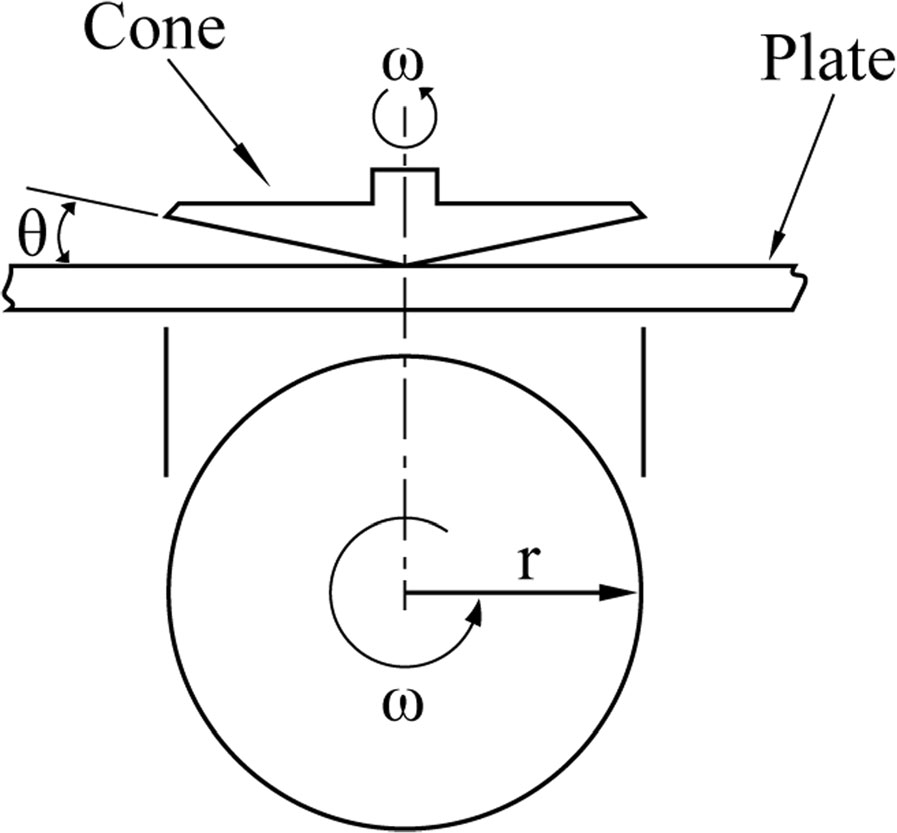
Schematic representation of a cone and plate viscometer

In the cone and plate viscometer, a nearly flat cone (with cone angle between 0.8° and 3°) in close proximity to a plate forms a narrow gap where the liquid is contained (Fig. 3) [65]. This cone and plate spindle geometry requires a sample volume of only 0.5 to 2.0 mL and generates a constant shear rate at all locations under the cone at any given rotational speed. The viscosity can easily be determined from shear stress (the torque) and shear rate (the angular velocity) by the following equation:

4 $$ \eta =\frac{3M\sin \theta }{2\pi {r}^3\omega } $$

where *M* is the torque input by the instrument, *θ* is the cone angle, *r* is the cone radius, and *ω* is the angular velocity.

Furthermore, both the elastic and viscous characteristics of the material can be studied by using a strain-controlled cone and plate rheometer [72, 73]. In this type of device, a motor is designed to impose a sinusoidal strain γ(t) to a material, and the resultant stress σ(t) is measured with a transducer. The phase angle (δ) between stress and strain is used to decompose the measured stress into an in-phase component and to determine the elastic modulus *G*^′^ of a material, defined as the in-phase stress divided by the amplitude of the strain, ${G}^{\prime }=\frac{\hat{\sigma}}{\hat{\gamma}} cos\delta$, where $\hat{\sigma}$ and $\hat{\gamma}$ are the amplitudes of the stress and strain, respectively. Similarly, the viscous modulus *G*^′′^ of the material is defined as the out-of-phase stress divided by the amplitude of the strain, ${G}^{\prime \prime }=\frac{\hat{\sigma}}{\hat{\gamma}} sin\delta$.

The cone and plate viscometer has been widely employed in the measurement of the rheological properties of airway surface liquid [26, 64, 66, 74]. As a result of measurements taken with a cone and plate viscometer, Baldry and Josse showed that comparable readings could be obtained with duplicate sputum samples at different shear rates [66]. Lieberman found that sputum viscosity could reach a relatively steady state after a limited amount shearing in a cone and plate viscometer [74]. Similarly, King et al. investigated the bulk shear viscosities of aqueous dispersions of calf lung surfactant in a cone and plate viscometer, which showed that the lung surfactant exhibited a complex non-Newtonian behaviour, with higher viscosities at low shear rates [75].

### Magnetic microrheometer

The magnetic micrcorheometer involves a pair of permanent magnets or electromagnets for generating a rotating magnetic field [13, 76–78]. The test fluid sample is placed in a small test tube with a concave and clear bottom. A metal microsphere is inserted in the sample. The tube, sealed to prevent evaporation of the sample, is centred between the two magnets. The rotating magnetic field generates a magnetic driving force that rotates the metal sphere. In the case of low frequencies and small sphere diameters, the sphere inertia can be neglected. Therefore, the angular speed of the sphere is determined by the rotational speed and strength of the magnetic field as well as the viscosity of the sample around the sphere. The motion of the sphere is monitored by a high-resolution video microscope set below the sample cell. The torque exerted on the sphere is proportional to the difference between the angular velocity of the magnetic field *Ω*_*B*_ and that of the sphere *Ω*_*S*_, and the shear rate of the flow is linearly proportional to *Ω*_*S*_. Thus, the viscosity of the test sample can be calculated depending on the shear rate by measuring *Ω*_*S*_ at different values of *Ω*_*B*_ [78]. In another type of displacement magnetic microrheometer, a micron-sized magnetic bead is carefully positioned in the sample and oscillated by means of an electromagnet at a variable frequency (ω) and amplitude [16, 79, 80]. Images of the bead are captured by a charge-coupled device (CCD) camera to measure bead displacement. The amplitude of the displacement of the bead and its phase shift with respect to the magnetic force are determined to calculate the elastic modulus *G*^′^ and viscous modulus *G*^′′^ of the sample using:

5 $$ {G}^{\prime }=\frac{f_0}{6\pi a{X}_0\left(\omega \right)}\cos \varphi \left(\omega \right) $$

6 $$ {G}^{\prime \prime }=\frac{f_0}{6\pi a{X}_0\left(\omega \right)}\sin \varphi \left(\omega \right) $$

where *f*_0_ is the amplitude of the applied force, a is the radius of the bead, *X*_0_(ω) is the frequency-dependent amplitude of the bead displacement, and φ(ω) is the frequency-dependent phase shift between the force and the bead displacement.

The two remarkable features of the magnetic microrheometer are the need for only microlitre quantities of sample volume and freedom from contamination. Consequently, it is well suited to the investigation of the rheological properties of lung fluids partially because only small lung fluid samples can be obtained in normal or disease conditions. King and coworkers pioneered the use of a magnetic rheometer to determine the viscoelastic properties of normal tracheal mucus from canines and discussed the significance of these rheological behaviours in terms of the clearance of secretions from the lung [77].

### Particle tracking microrheometer

Particle tracking microrheology can be used to characterize the linear viscoelasticity of complex fluids with the accuracy of bulk rheology measurements but with smaller sample volumes on the order of picolitres to microlitres required [13, 81, 82]. A modern experimental set up to perform particle tracking microrheology experiments primarily consists of a light source, a colloidal probe, optical microscopy, a fast COMS camera, and specialized particle tracking software.

Colloidal spheres are embedded into a soft viscoelastic fluid, and movies are made of the Brownian motion of the colloidal probes in the sample using the fast COMS camera. The positions of the centroids of the colloidal probes are subsequently matched frame by frame using a specialized routine to identify each particle and generate its trajectory. Then, the mean squared displacements (MSD),〈*Δr*^2^(*t*)〉, of individual particles are calculated from the colloidal sphere trajectories.

Mathematical analysis of MSD can provide a measure of the linear viscoelasticity of the test fluid as a function of time or frequency. The simplest method, for example, is to calculate the creep compliance *J*(*t*) in the form [81–84]:

7 $$ J(t)=\frac{\pi a}{kT}\left\langle \varDelta {r}^2(t)\right\rangle $$

where *kT* is the thermal energy and *a* is the radius of the particle. A purely viscous liquid of shear viscosity *η*, such as water or glycerol, subjected to a constant stress, exhibits a creep compliance that increases linearly with time, *J*(*t*) = *t*/*η*; a highly elastic material of modulus *G*_0_ under stress exhibits a time-independent compliance *J* = 1/*G*_0_; the time-dependent compliance of a viscoelastic material such as mucus or cytoplasm shows an intermediate behaviour [13, 83].

In fact, people often prefer to work with the complex shear modulus (G^∗^(ω) = G^′^(ω) + iG^′′^(ω)) since its real and imaginary parts more clearly define the contributions of elasticisty (G^′^) and dissipation (the viscosity, η = G^′′^/ω as ω → 0) to the viscoelasticity response [81]. The G^∗^(ω) of the complex fluids can be obtained from measurements of the time-dependent mean square displacement, 〈*∆*r^2^(t)〉, of thermally driven colloidal spheres suspended in the fluid using a generalized Stokes-Einstein (GSE) equation [85]. The frequency-domain representation of the GSE equation takes the following form:

8 $$ {\mathrm{G}}^{\ast}\left(\upomega \right)=\frac{\mathrm{kT}}{\uppi \mathrm{a}\mathrm{i}\upomega \mathrm{Fu}\left\{\left\langle \Delta  {\mathrm{r}}^2\left(\mathrm{t}\right)\right\rangle \right\}}=\frac{\mathrm{kT}}{6\uppi \mathrm{a}{\mathrm{D}}^{\ast}\left(\upomega \right)} $$

where D is the time-dependent diffusion coefficient and Fu is the Fourier transform. Consider spheres diffusing in a purely viscous fluid or a viscoelastic material; 〈*∆*r^2^(t)〉 can be calculated by Eq. (9) and (10), respectively,

9 $$ \left\langle \Delta  {\mathrm{r}}^2\left(\mathrm{t}\right)\right\rangle =6\mathrm{Dt} $$

10 $$ \left\langle \Delta  {\mathrm{r}}^2\left(\mathrm{t}\right)\right\rangle ={\mathrm{r}}_0^2\left[1-\exp \left(-6\mathrm{Dt}/{\mathrm{r}}_0^2\right)\right] $$

where ${\mathrm{r}}_0^2$ is the saturation value of the mean square displacement of the spheres as time approaches infinity. Combining Eq. (8) with (9) or (10), the frequency-independent viscosity is recovered $\upeta =\frac{\mathrm{kT}}{6\uppi \mathrm{aD}}$.

Dawson et al. used multiple particle tracking to determine the effective viscoelastic properties of human cystic fibrotic sputum at the micron scale. They found that CF sputum microviscosity was an order of magnitude lower than its macroviscosity, suggesting that the enhanced viscoelasticity of CF sputum correlates with the increased microheterogeneity in particle transport [28]. A primary problem with a particle tracking micorheology-based characterization of airway mucus is a possible overestimation of the true mucus viscoelasticity due to adhesive interactions between colloidal probes and mucus. In addition, the maximally achievable viscosity and shear rate ranges are limited due to restrictions on particle sizes and the temporal resolution of tracking, respectively.

## Methods for measuring surface tension of airway surface liquid

Over the past few decades, a variety of measuring techniques have been developed for determining the surface activity of surfactant materials derived from the lung. Among these are film balances, bubble methods and drop shape analysis methods. In addition, the surface tension of pulmonary surfactant can be inferred from pressure-volume data. In the following section, we will discuss classical methods and recent techniques in terms of their basic principles, advantages and limitations to help research workers select the method(s) best suited to their needs.

### The Langmuir-Wilhelmy balance

Clements first introduced the Langmuir-Wilhelmy surface balance to determine the surface tension-area relationship in his pioneering studies of lung extracts [86, 87]. In this method, lung extracts are dropped onto the surface of a subphase substance (usually normal saline) contained in the trough of the surface balance, while the exposed surface area is varied over a wide range by means of a movable barrier. A roughened and clean platinum plate is attached to a balance with a thin metal wire. When the plate is perpendicularly dipped into the liquid, the downward force (*F*) on it due to wetting is measured by a balance connected to a transducer. The surface tension (γ) can then be calculated using the following equation:

11 $$ \upgamma =\frac{F}{L\bullet \cos \left(\theta \right)} $$

where *L* is the wetted perimeter of the plate and θ is the contact angle between the liquid phase and the plate. In practice, the platinum plate is assumed to be completely wetted, and therefore, the contact angle is 0°. In this way, Clements and Brown showed that the surface tension of a lung-derived surface approached a lower limiting of 10~15 dyn/cm and an upper limiting of 40~50 dyn/cm [86, 88].

Although the Langmuir-Wilhelmy balance is one of the most commonly used tools for measuring surface tension, there are still some drawbacks to this apparatus in terms of investigating the tension-area behaviour of lung extracts. One of the most intractable problems has been the film leakage that occurs on the surfaces of the restraining walls and barrier, causing experimental artefacts [89]. Furthermore, large sample volumes are required on the Langmiur-Wilhelmy surface balance because of its large size. Finally, the apparatus does not seem readily adaptable to rapid oscillations of surface area at rates corresponding to a normal cycle of breathing [57].

### Captive bubble method

**Fig. 4:**
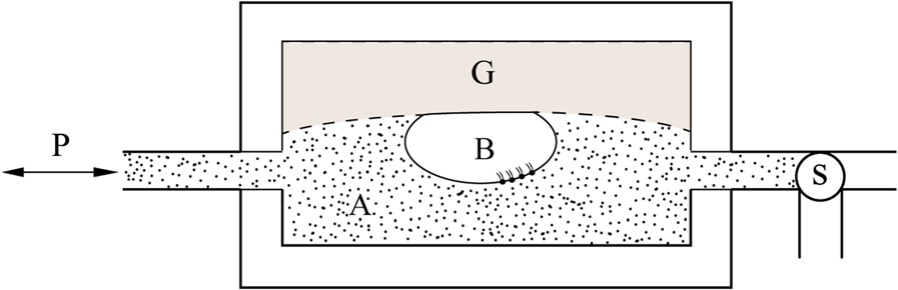
Captive bubble chamber. A lung surfactant suspension (A) is placed into a glass flow-through chamber, and a captive bubble (B) is formed by a syringe within the aqueous phase and then allowed to float against the agarose gel (G) ceiling. After the stopcock (S) is closed, B can be compressed or expanded by withdrawing fluid through the pressure control port (P) [94]

The captive bubble method developed by Schürch and coworkers is useful for reproducing the in situ behaviour of lung surfactant monolayers because it eliminates the possibility of surface film leakage [90–93]. In this method, a lung surfactant suspension is placed into a glass flow-through chamber, and a bubble of atmospheric air is introduced and allowed to float against the slightly concave hydrophilic agarose ceiling of the chamber (Fig. 4) [90, 94]. The bubble volume is compressed and expanded by varying the fluid pressure in the chamber. From the beginning of adsorption measurements, the chamber pressure is rapidly reduced to an estimated level at which the bubble just doubles its diameter from 2~3 mm to 4~6 mm. Then, the captive bubble is submitted to a number of compression-expansion cycles. As the pressure is increased, the bubble volume and surface area are decreased, compressing the absorbed surfactant monolayer at the air-water interface, during which the bubble progressively flattens, indicating a lower surface tension.

Images of the bubble are recorded by a computer digital image system. By measuring the height and diameter of the bubble in the video picture, the surface tension and area can be calculated using the approach of Malcolm and Elliott or the formulas of Rotenberg [94]. One important feature of the captive bubble approach is that low and stable surface tensions of 1~2 mN/m can be obtained upon the first quasi-static or dynamic compression following adsorption, and therefore, it is suitable for studies on the role of surfactant proteins in surfactant formation [90, 94].

### Microdroplet method

Schürch et al. reported the use of the microdroplet method to measure alveolar surface tension directly in an excised lung [92, 95–97]. The method is based on the observation that a droplet of a nonpolar test liquid resting on top of a monolayer at the air-water interface changes its shape from a sphere to a thin lens as the interfacial tension is raised beyond the value characteristic of the test fluid. In a prior calibration experiment, test fluid droplets with known surface tension are placed onto a surfactant film on a fluid substrate in the surface balance. The shape of the droplets is changed by varying the surface tension of the film and is monitored with a microscope. The relative diameter of the lens (defined as the diameter normalized to its spherical shape) can thus be plotted as a function of the film surface tension. Conversely, if the relative diameter is known, the film surface tension can be determined with this calibration curve.

In the subsequent measurements of alveolar surface tension in situ, the alveolus is punctured by a micropipette with a tip diameter of 1~2 μm, and then a test fluid droplet of fluorocarbon liquid or silicone oil is deposited onto the alveolar surface. The diameter of the spherical drop from the pipette before deposition is usually 10~100 μm [95]. After deposition onto the alveolar surface, the drop spreads out into a lens shape whose diameter depends on the alveolar surface tension. By using the microdroplet method, Schürch et al. confirmed that the surface tension in the excised rat lungs at functional residual capacity was between 1 and 10 mN/m [97] and further showed that the maximum surface tension at TLC was approximately 30 mN/m [96]. Most importantly, Schürch et al. found that in excised cat lungs at a given volume between 40 and 70% of TLC, no difference in surface tension could be detected with respect to alveolar size and location [95].

### Pendant drop method

The pendant drop method is extensively used for surface tension measurement, even at elevated temperatures and pressures [98–101]. In the pendant drop setup, a drop is formed at the tip of a stainless-steel needle. The volume and surface area are controlled by moving the plunger of the syringe connected to a stepper motor. The pendant drop is constrained inside a glass cuvette to maintain controllable environmental conditions (i.e., temperature and pressure). The drop images are magnified by a horizontally mounted microscope and then acquired using an automatic camera. Then, these images are digitalized and stored in a computer for further extraction of the pendant drop profile to calculate the surface tension [102, 103].

**Fig. 5:**
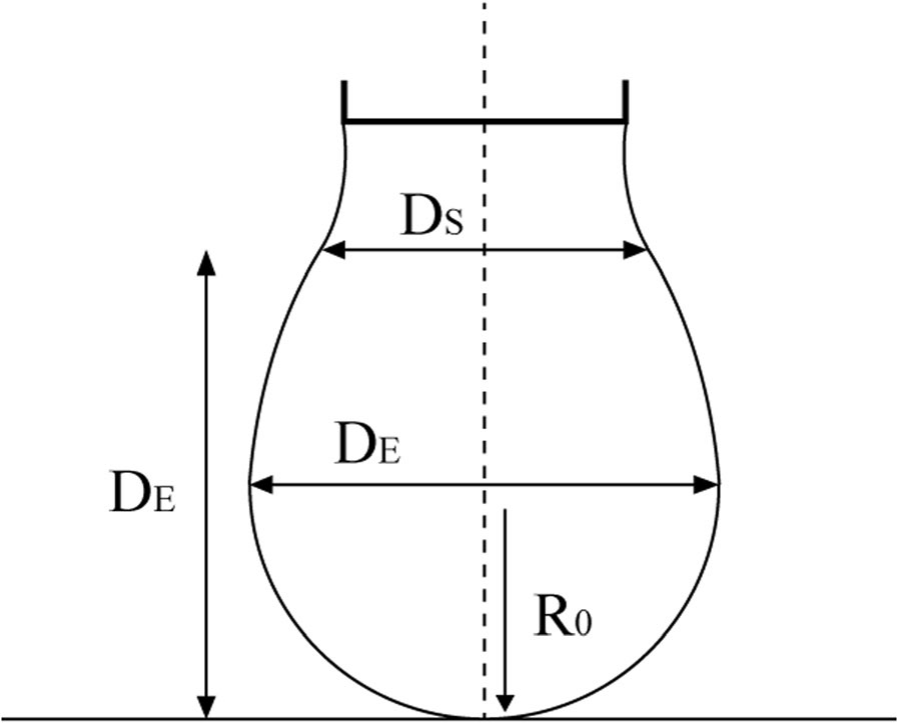
Geometry of the pendant drop method

In the traditional method, the maximum diameter D_E_ is measured from the drop profile and the diameter D_S_ at a horizontal plane at a distance D_E_ from the bottom of the drop (Fig. 5), after which the surface tension is calculated by the equation

12 $$ \upgamma =\Delta  \uprho \mathrm{g}{D}_E^2/H $$

where ∆ρ is the density difference between the drop and the surrounding medium, g is the gravitational acceleration, and H is a function of S = *D*_*S*_/*D*_*E*_, which can be read from a table [99, 104]. Axisymmetric drop shape analysis (ADSA) has recently been developed as a more accurate and applicable technique for measuring surface tension based on acquired drop images. Briefly, ADSA matches the experimental drop profile through an optimization procedure to a theoretical profile calculated from the Laplace equation of capillarity. The matched theoretical drop profile is used to find the surface tension of the pendant drop [99, 100, 102, 103].

The pendant method offers several distinct advantages over the conventional film balance, including a high degree of automation, a small sample size, and the ease with which the sample can be isolated from the environment to protect it from contamination [89, 100]. Since the pendant drop method does not suffer a similar restriction in compression rate compared with the Langmuir-Wilhelmy surface balance, it has been used to examine the rate dependence of the surface pressure-surface area isotherm of a DPPC monolayer. The results obtained by the pendant drop method are in good agreement with those from the conventional film balance measurements [103, 105].

### Pressure-volume measurements

Some early studies used pressure-volume data from air- and liquid-filled excised lungs to calculate alveolar surface tension [106–110]. For example, assuming a maximum surface tension and a constant relationship between lung surface area and lung volume, Bachofen and coworkers [110] derived an equation that relates the surface tension (γ) to lung volme (V) and the component of recoil pressure (P_s_) caused by surface tension in the form

13 $$ \upgamma =\frac{3}{2\mathrm{k}}{\mathrm{P}}_{\mathrm{s}}{\mathrm{V}}^{1/3} $$

where k is the shape factor, which can be found from the maximum surface tension. Thus, for each pair of air and saline P-V curves, the corresponding surface tension-surface area can be obtained by the above equation. Smith and Stamenovic [109] provided another approach to deduce values of alveolar surface tension from pressure-volume data. Their approach rests on the assumption that at a given volume, P_s_ is uniquely determined by γ. First, the pressure-volume curves of control air-filled lungs and test liquid-filled lungs with a limited set of fixed interfacial tensions are measured. As the intersections between the curves with the normal and test liquid interfaces define states of equal surface tension, by pooling data from all intersections, the surface tension-lung volume relationships are obtained. In addition, Wilson used an energy analysis method to calculate the values of surface tension from pressure-volume loops. The reader is referred to reference [108] for a detailed description.

## Conclusions

In recent years, measuring the rheological properties of airway surface liquid has attracted considerable clinical attention due to new developments in microrheology instruments and methods. The quantitative characterization of the viscoelasticity and surface tension of airway surface liquid contributes to a better understanding of physiological processes such as airway mucus trapping and clearance and ciliary action to further identify potential markers for ranking the severity of relevant muco-obstructive lung diseases and to develop muco-inert nanoparticle systems for improved drug and gene delivery to mucosal tissues; a good knowledge of lung surfactant dynamics to improve surfactant replacement therapy for respiratory distress in neonates and even adults; and a deep insight into the micromechanical mechanisms of VILI to prescribe personalized mechanical ventilation in ARDS patients. In terms of measurements of viscosity and surface tension of airway surface liquid, the cone and plate system is currently the most commonly used instrument for determining bulk viscosity in clinical practice, while multiple particle tracking is more suitable for probing the microviscosity of lung fluids. In light of the use of the axisymmetric drop shape analysis algorithm and the rapid development of data acquisition and image processing techniques, pendant drop methods have seen a broad prospective application in the surface tension measurement of airway surface liquid.

## Data Availability

Not Applicable.

## Abbreviations

ADSA: Axisymmetric drop shape analysis
ALI: Acute lung injury
ARDS: Acute respiratory distress syndrome
CCD: Charge-coupled device
CF: Cystic fibrosis
COPD: Chronic obstructive pulmonary disease
DPPC: Dipalmitoyl phosphatidyl choline
FRC: Functional residual capacity
GSE: Generalized Stokes-Einstein equation
HBE: Human bronchial epithelial
INSURE: INtubtion-SURfactant-Extubation
MSD: Mean squared displacement
NRDS: Neonatal respiratory distress syndrome
PEG: Polyethylene glycol
PS: Pulmonary surfactant
SP: Surfactant protein
SRT: Surfactant replacement therapy
TLC: Total lung capacity
VILI: Ventilator-induced lung injury
